# Effect of JAK-STAT pathway in regulation of fatty liver hemorrhagic syndrome in chickens

**DOI:** 10.5713/ajas.19.0874

**Published:** 2020-02-25

**Authors:** Yaling Zhu, Huirong Mao, Gang Peng, Qingjie Zeng, Qing Wei, Jiming Ruan, Jianzhen Huang

**Affiliations:** 1 College of Animal Science and Technology, Jiangxi Agricultural University, Nanchang, 330045, China; 2 Department of Pathophysiology, Anhui Medical University, Hefei 230000, China; 3 Laboratory Animal Research Center, College of Basic Medical Science, Anhui Medical University, Hefei 230000, China

**Keywords:** Fatty Liver Hemorrhagic Syndrome, RNA-Seq, JAK-STAT Pathway, Differentially Expressed Genes, Chicken

## Abstract

**Objective:**

To explore the molecular mechanisms of fatty liver hemorrhagic syndrome (FLHS) in laying hens, an experiment was conducted to reveal the differences in histopathological observation and gene expression between FLHS group and normal group.

**Methods:**

We compared the histopathological difference using hematoxylin and eosin staining and proceeded with RNA sequencing of adipose tissue to search differentially expressed genes and enriched biological processes and pathways. Then we validated the mRNA expression levels by real-time polymerase chain reaction and quantified protein levels in the circulation by enzyme-linked immunosorbent assay.

**Results:**

We identified 100 differentially expressed transcripts corresponding to 66 genes (DEGs) were identified between FLHS-affected group and normal group. Seven DEGs were significantly enriched in the immune response process and lipid metabolic process, including phospholipase A2 group V, WAP kunitz and netrin domain containing 2, delta 4-desaturase sphingolipid 2, perilipin 3, interleukin-6 (*IL-6*), ciliary neurotrophic factor (*CNTF*), and suppressor of cytokine signaling 3 (*SOCS3*). And these genes could be the targets of immune response and be involved in metabolic homeostasis during the process of FLHS in laying hens. Based on functional categories of the DEGs, we further proposed a model to explain the etiology and pathogenesis of FLHS. *IL-6* and *SOCS3* mediate inflammatory responses and the satiety hormone of leptin, induce dysfunction of Jak-STAT signaling pathway, leading to insulin resistance and lipid metabolic disorders. Conversely, *CNTF* may reduce tissue destruction during inflammatory attacks and confer protection from inflammation-induced insulin resistance in FLHS chickens.

**Conclusion:**

These findings highlight the therapeutic implications of targeting the JAK-STAT pathway. Inhibition of *IL6* and *SOCS3* and facilitation of *CNTF* could serve as a favorable strategy to enhance insulin action and improve glucose homoeostasis, which are of importance for treating obesity-related disorders for chickens.

## INTRODUCTION

Fatty liver hemorrhagic syndrome (FLHS) is a lipid metabolism disorder, which is mostly observed in commercial laying hens. FLHS causes a dramatic drop in egg production, even leads to an increase in mortality and results in considerable economic losses in the poultry industry [1]. For FLHS affected hens, the most obvious changes are enlarged and yellowish, putty-colored livers with various degrees of hemorrhages and an excessive amount of abdominal fat. Several factors have been implicated in the cause of FLHS, including nutrition, environment, heredity, toxic substances and hormones [2,3]. The major cause of FLHS is nutrition, as 97% of the affected birds were found to have large fat depots or were obese [4]. In modern poultry industry, an excessive intake of dietary energy is the major cause of FLHS. Recently, a high-energy low-protein (HELP) diet induced FLHS model has been successfully established [5].

The metabolic disorders such as obesity and liver steatosis often result from the disruption of lipid homeostasis and overactivation of immune pathways. And these damages are commonly localized around expanding volumes of adipose tissue, and in particular visceral adipose tissue [6]. Adipose tissue is an important endocrine organ that controls energy hemostasis by storing excess energy in the form of triacylglycerol (TAG) and releasing energy in the form of free fatty acid. Excessive lipid storage in adipose tissue results in obesity and ultimately its related complications including insulin resistance, non-alcoholic fatty liver disease and other lipid metabolic diseases [7]. Alternatively, adipose tissue is currently considered as a hormonally active system in the control of metabolism, not only as a store of excess energy. It has been accepted there is a close link between chronic low-grade inflammation in adipose tissue and metabolic disorders [8]. Accumulated evidence indicates that adipose tissue can release hormones and adipokines which affect glucose homeostasis, lipid metabolism, and inflammation. For example, interleukin-6 (*IL-6*) is a multifunctional cytokine that plays a major role in regulating immune responses, acute phase reactions and hematopoiesis in chicken and human [9]. An adipocyte-derived hormone of leptin can activate the JAK-STAT signaling pathway through the chicken leptin receptor (chLEPR) to regulate energy homeostasis [10], and suppressing cytokine signaling 3 (*SOCS3*) plays an essential role in affecting systemic inflammation, insulin resistance, and leptin resistance [6]. Thereby, a better understanding of the endocrine function of adipose tissue may shed insights into the lipid metabolic disorders and the importance of inflammation as a pathogenic pathway in the development of many hallmarks of the metabolic syndrome including insulin resistance, leading to more rational therapy for these increasingly prevalent disorders.

To date, as far as we know, systematic studies to detect the transcriptomic changes in abdominal adipose tissue from FLHS affected (experimental) hens in comparison with healthy (control) individuals are rare. To this end, we explored the RNA sequencing technology to compare gene expression profiling in the abdominal fat tissue from FLHS experimental and control chickens, and to identify candidate genes and pathways functionally related to FLHS in chickens.

## MATERIALS AND METHODS

### Ethics and consent

All the tested animals are raised in compliance with the care and use guidelines of experimental animals established by the Ministry of Agriculture of China. This study was approved by the ethics committee of Jiangxi Agricultural University (Approval number: JXAULL-20190019).

### Experimental animals and tissue collection

All animal experiments were approved by the Jiangxi Agricultural University Animal Care and Use Committee. Ninety healthy 155-day-old Hyline Brown layers with an average body weight of 1.5 kg were chosen in this study. All layers were acclimatized for 7 days before the start of the experiment. These layers were randomly divided into the experimental and control groups, and each group contained 45 layers with 3 replicates. These chickens were fed the same basal diet that was formulated to meet the nutritional requirements of layers [11]. Layers in the control group were fed basal diet, and layers in the experimental group were fed ‘HELP’ diet (Supplementary Table S1). On experimental day 60, the livers were harvested from the tested chickens after they were sacrificed humanely. Portions of the fresh liver samples were stored at −80°C until further use.

### Histopathological examination

Fresh liver samples were dissected, rinsed with saline and fixed in 10% neutral buffered formalin. The subcutaneous fat was immersion-fixed in 4% formaldehyde solution for 24 h, then dehydrated and embedded in paraffin using a standard procedure [12]. The formalin-fixed samples were routinely processed, embedded in paraffin, stained with hematoxylin and eosin (H&E). The stained sections were observed using an optical microscope, and photographs were taken. Diameters of adipocyte staining were determined using Image-Pro Plus 6.0 software.

### Statistical analyses

Phenotypic values were presented as mean±standard deviation (mean±SD). Statistical comparisons of phenotypic values between the experimental and control groups were conducted by Student t-test. The statistical difference was considered as significant at p≤0.05 and highly significant at p≤0.01.

### RNA extraction and sequencing

Total RNA was extracted from 100 mg of adipose tissue from three FLSH experimental individuals and three control individuals using the RiboPure kit (Ambion, Austin, TX, USA) according to the manufacturer’s protocol. RNA integrity was assessed by an Agilent Bioanalyser 2100 and RNA Nano 6000 Lab chip kit (Agilent Technologies, Santa Clara, CA, USA). Sequencing libraries were generated using the NEBNext Ultra Directional RNA Library Prep Kit (Illumina, San Diego, CA, USA) following the manufacturer’s recommendations, and index codes were added to attribute sequences to each sample. Then the paired-end sequencing of the libraries was constructed on a Hi-Seq 4000 platform (Illumina, USA) via Novogene (Novogene, Beijing, China).

### Mapping, assembling and annotation of sequence reads

First, the RNA-seq reads were discriminated based on the indexing adaptors. Low-quality reads and those containing poly-N were then removed from raw data using FastQC v0.11.7 (http://www.bioinformatics.bbsrc.ac.uk/projects/fastqc). Next, the filtered reads were mapped against the chicken reference genome Gallus_gallus-5.0 (Ensembl) using STAR-2.5.3a, a fast splice junction mapper for short and long RNA-seq reads to a reference genome using uncompressed suffix arrays. Parameters of STAR were set to allow only unique alignment to the reference genome. Transcripts were assembled and quantified by Stringtie-1.3.3b [13]. In addition, we explored S-MART (http://urgi.versailles.inra.fr/Tools/S-MART) to calculate the distribution of reads mapped to exons, introns and 1 kb upstream/downstream of the annotated genes. To count the number of reads that uniquely mapped to an exon, featureCounts was used with ‘gene’ as feature and not strand-specific [14]. Since low expressed genes are more vulnerable to measurement errors, we removed low expressed genes whose counts were lower than 2 in 90% samples. And then FPKM (expected number of Fragments Per Kilobase of transcript sequence per Millions base pairs sequenced) of each gene was calculated based on the length of the gene and read count mapped to this gene. FPKM considers the effect of sequencing depth and gene length for the read counts at the same time, and is currently the most commonly used method for estimating gene expression levels from RNA-seq data [15].

### Differential gene expression analyses

Differential expression analyses of the FLHS experimental and control groups was performed using the DESeq2 R package [15]. It provides statistical routines for determining differentially expressed genes (DEGs) from digital gene expression data using a model based on the negative binomial distribution. The resulting p-values were adjusted using the Benjamini and Hochberg’s approach for controlling the false discovery rate. Genes with adjusted p-values (Padj) of less than 0.05 and fold changes of greater than 1.2 were assigned as DEGs.

### Gene ontology and pathway enrichment analyses

DAVID (https://david-d.ncifcrf.gov/) and PANTHER (http://www.pantherdb.org/) were executed to identify over-represented gene ontology (GO) terms and pathways of the 66 DEGs with a log2(fold change) higher than 1.2 and Padj (false discover rate) less than 0.05 between control and FLHS chickens. GO terms with corrected p-value less than 0.05 were considered significantly enriched by DEGs. Kyoto encyclopedia of genes and genomes (KEGG) is a database resource for understanding high-level functions and utilities of the biological system, such as the cell, the organism and the ecosystem, from molecular-level information, especially large-scale molecular datasets generated by genome sequencing and other high-through put experimental technologies (http://www.genome.jp/kegg/). We used the KOBAS software (http://kobas.cbi.pku.edu.cn) to test the statistical enrichment of DEGs in KEGG pathways.

### Quantitative real-time polymerase chain reaction

RNA was reverse-transcribed into first-strand cDNA with Moloney Murine Leukemia Virus transcriptase (Promega, Madison, WI, USA) and oligo (dT) (TaKaRa, Purwokerto, Japan) using 2 μg of total RNA. The expression of *IL-6*, *SOCS3*, and ciliary neurotrophic factor (*CNTF*) genes were quantified by real-time polymerase chain reaction (RT-PCR) using a LightCycler 480 instrument with the LightCycler 480 SYBR Green I Master Mix (Roche, Santa Clara, CA, USA). For RT-PCR amplification, cDNA was pre-denatured at 95°C for 10 min, followed by 40 cycles of 95°C for 30 s, and 60°C for 1 minute. The relative expression level of the target gene was normalized to that of the housekeeping gene β-actin by the 2^−ΔΔCT^ method.

### Enzyme-linked immunosorbent assay

Blood samples were collected from healthy group and FLHS group respectively, and sera were separated by centrifugation at 1,000×g for 20 minutes and the supernatants carefully collected and stored at −20°C until use. The enzyme-linked immunosorbent assay (ELISA) of IL-6, SOCS3, and CTNF used the Chicken Interleukin 6 ELISA Kit (Catalog #MBS 037319), chicken suppressor of cytokine signaling 3 ELISA Kit (Catalog #OKEH03978) and chicken ciliary neurotrophic factor ELISA Kit (Catalog #MBS260527) respectively. The detailed protocols were obtained from https://cdn.mybiosource.com/ and https://www.biocompare.com/. ELISA results were determined for each serum sample in duplicate. Cut-off point of an experimental sample was set to be at least two times higher than that of the control sample.

## RESULTS AND DISCUSSION

### Pathological and histopathological observation of the liver and adipose tissues from experimental and control individuals

**Figure 1:**
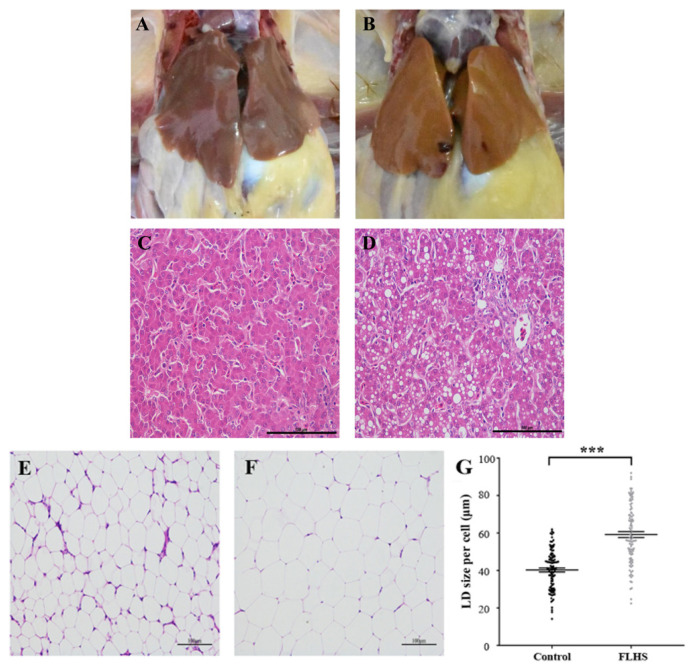
Anatomical and histopathological images of the liver in chickens. (A) Livers of normal chickens; (B) Livers of fatty liver hemorrhagic syndrome (FLHS) affected chickens; (C) Histopathological observation of the liver in normal chickens. (D) Histopathological observation of the liver in FLHS affected chickens. Scale bars = 100 μm; (E–F) Subcutaneous fat sections stained with haematoxylin and eosin from control (E) and experimental animals (F). Scale bars = 100 μm. (G) Based on sections in (E) and (F), the sizes of lipid droplets (LDs) were calculated from 100 adipocytes in each group. LDs in FLHS chickens were significantly larger than those in control chickens (p<0.001).

Compared with healthy hens (Figure 1A), FLHS affected individuals showed large, friable and soft livers that varied in color from yellow to orange (Figure 1B), which is consistent with previous reports [1,10]. As to the difference of biochemical parameters between these two groups, we found that there was no statistical difference in body weight of hens, but the liver index in the experimental group was significantly (p< 0.01) higher than that in the control group. There were similar change trends for (appear in) the hepatic triglyceride (TG) and total cholesterol (TC) (Table 1) i.e. the concentration of hepatic TG and TC in experimental individuals were much higher (p<0.01) than that in control individuals. As revealed by the H&E staining, FLHS affected animals also had pathological changes in the liver, including lesions and micro-vesicular fatty change (liver cells were filled with multiple fat droplets) (Figure 1D), although they displayed regular hepatic sinusoids and normal hepatic cords (Figure 1C).

To confirm that lipid accumulation in adipose tissue was enhanced in FLHS affected chickens, we performed microscopic examinations on the adipose tissue from the two groups of animals. We found that adipocytes were smaller and more compact in the adipose tissue of normal chickens (Figure 1E) relative to FLHS affected chickens (Figure 1F). Similarly, the sizes of lipid droplets in FLHS affected chickens were significantly bigger (p<0.001) than those in normal broiler chickens (Figure 1G).

### Mapping and annotation of RNA sequencing reads

We obtained about 40 million (M) of 150bp paired-end reads for each sample (Supplementary Figure S1A). After ambiguous mapping (allowing for multi-hits) via STAR-2.5.3a [16], a total of ~36 M reads for each sample were mapped against the chicken reference genome Gallus_gallus-5.0 (Ensembl), and only ~4 M were classified as improper pairs (Supplementary Figure S1B). Among the mapped reads, 80% of these reads were mapped to exonic regions, 14% to intergenic regions, and 6% to intronic regions (Supplementary Figure S1B). The rest (4 M) fell into either one of these categories: reads without a mapped mate pair or mate was multiple mapped. One reasonable explanation of the amount of improperly mapped reads is the poor quality of the chicken genome assembly. In addition, any situation where the distance between the mates is larger than the confidence interval of the insert size distribution could be interpreted as trans-splicing events, structural variants or simply mapping artifacts [17].

### Identification of differentially expressed genes between experimental and control chickens

**Figure 2:**
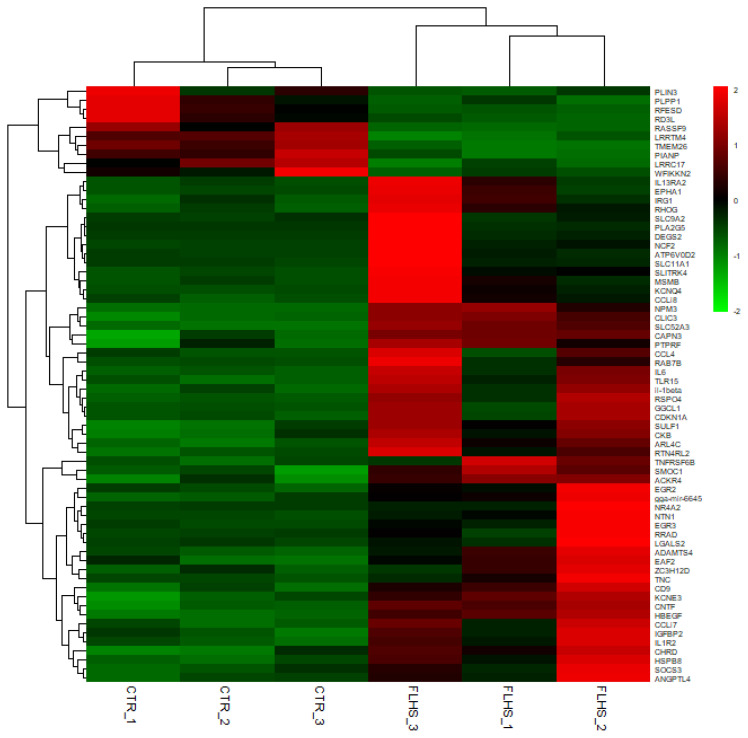
Transcription profiles of 66 differentially expressed genes between healthy chickens and FLHS affected chickens. CTR, control; FLHS, fatty liver hemorrhagic syndrome.

In total, 100 differentially expressed transcripts corresponding to 66 genes (DEGs) were identified between experimental and control animals with a log2(fold change) higher than 1.2 at Padj (false discover rate) <0.05 (Supplementary Figure S2). Among these transcripts, 83 were up-regulated and 17 were down-regulated in FLHS affected animals (Supplementary Table S2). The top ten up- and down-regulated genes with higher log2(fold change) value are listed in Table 2, including phospholipase A2 group V (*PLA2G5*) [18], delta 4-desaturase sphingolipid 2 (*DEGS2*) [19], *IL-6* [20], solute carrier family 9 member a2 (*SLC9A2*) [21], wap kunitz and netrin domain containing 2 (*WFIKKN2*) [22], perilipin 3 (*PLIN3*) [23], alpha associated neural protein (*PIANP*) [24], phospholipid phosphatase 1 (*PLPP1*) [25], which have been reported to be involved in the regulation of inflammatory system process and lipid metabolic process. Given the unknown function of the 34 novel transcripts, we proceeded with hierarchical clustering of 66 annotated DEGs to show the differential gene expression profile between normal and FLHS affected chickens (Figure 2).

### Gene annotation and gene ontology analyses of differentially expressed genes

**Figure 3:**
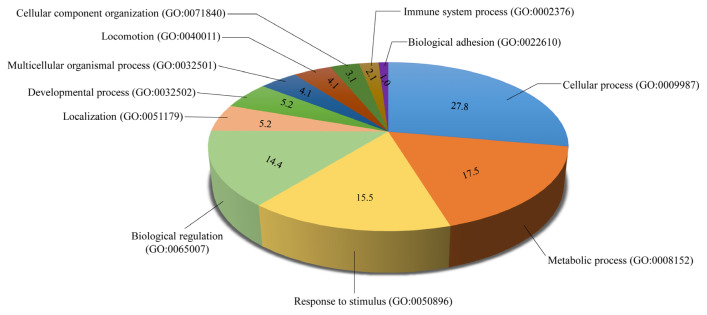
A pie chart of enriched biological processes involving 66 differentially expressed genes.

We first adopted PANTHER (http://www.pantherdb.org/) to understand gene ontology and function of the 66 DEGs in more detail. These genes are involved in multiple function terms, such as lipid metabolic process (GO: 0006629; *DEGS2*, *PLIN3*), fatty acid metabolic process (GO: 0006631; immune-responsive gene 1, phospholipid metabolic process (GO: 0006644; sulfatase 1, *PLA2G5*) and immune system process (GO: 0002376; *WFIKKN2*, solute carrier family 11 member 1 (*SLC11A1*) (Supplementary Table S3). These genes are also participated in multiple biological processes, such as “cellular process”, “metabolic process”, “response to stimulus”, “biological regulation”, “immune system process”, etc. (Figure 3), indicating that these processes could be triggered during the development of FHLS in chickens. Intriguingly, four genes including *DEGS2*, *PLIN3*, *PLA2G5*, and *WFIKKN2* exhibited the noteworthy log2(fold change) value with 5.91-fold, 1.87-fold, 7.03-fold, and 2.73-fold (p<0.01) respectively in the FLHS affected samples (Table 2) during the biology process of lipid metabolic process and immune system process, which were explored for further analyses. To further search for significantly overrepresented gene ontology terms involving 66 DEGs, functional annotation was also performed with the DAVID bioinformatics resources 6.7 (https://david-d.ncifcrf.gov/). Similar biological processes and interleukin regulation terms were significantly enriched (Supplementary Table S4), which was in accordance with the result of PANTHER. Besides, inflammatory related genes including *IL6*, *CNTF*, angiopoietin like 4, netrin 1, CD9 molecule, tenascin C, *SLC9A2*, *SLC11A1*, heparin binding EGF like growth factor, insulin like growth factor binding protein 2 were also over-represented in multiple GO terms (Supplementary Table S4, Figure S3).

Then according to general bioinformatics analyses and functional annotation results of our RNA-Seq data, six DEGs (*PLA2G5*, *WFIKKN2*, *DEGS2*, *PLIN3*, *IL-6*, and *CNTF*) were repeatedly over-represented in immune response process and lipid metabolic process (Supplementary Figure S3). Among these genes, *PLA2G5* showed the most striking fold change between experimental and control individuals, which has also been implicated in dysregulated lipid metabolism and low-grade inflammation [18]. *WFIKKN2* was the most significant down-regulated gene and over-represented in immune system process (GO: 0002376), which functions as a modulator of immunity [26]. In lymphocytes, a down regulation of *WFIKKN2* was evidenced in quaternary memory T cells as compared to primary memory T cells [27]. *DEGS2* (up-regulated) and *PLIN3* (down-regulated), were enriched in lipid metabolic process (GO: 0006629). *DEGS2* belongs to the desaturase/hydroxylase superfamily, which has a sphingolipid dihydroceramide hydroxylase activity and plays a role in signal transduction and cell apoptosis [19]. *PLIN3* is important for TAG storage and oxidation [23]. Loss-of-function mutations in *PLIN1* cause severe metabolic phenotypes, including impaired suppression of basal lipolysis, severe insulin resistance, type 2 diabetes, dyslipidemia and fatty liver disease [28]. *IL6*, significantly up-regulated in FLHS affected chickens, is known as both a pro-inflammatory cytokine and an anti-inflammatory myosin [20]. But in adipose tissue, as obesity related diseases like FLHS are always associated with hyper-activation of immune cells, pro-inflammatory cytokine *IL6* would impair insulin signaling indirectly by increasing serum none-sterified fatty acids, which have independently been shown to induce insulin resistance in multiple tissues [29]. In contrast, *CNTF*, a survival factor for various neuronal cell types, is relevant to reducing tissue destruction during inflammatory attacks and acts centrally by inducing hypothalamic neurogenesis to modulate food intake and peripherally by altering hepatic gene expression, in a manner similar to that of leptin (act on the hypothalamus to regulate food intake and energy expenditure), as well as reduces body mass and increases fat oxidation through activation of AMPK and restore insulin sensitivity [30].

Altogether, these results indicate that the six genes (*PLA2G5*, *WFIKKN2*, *DEGS2*, *PLIN3*, *IL6*, and *CNTF*) related to immune response process and lipid metabolic process could play a role in the development of FLHS in chickens. However, further investigations are required to establish the role of these genes in chicken FLHS.

### Differentially expressed genes over-represented pathways

**Figure 4:**
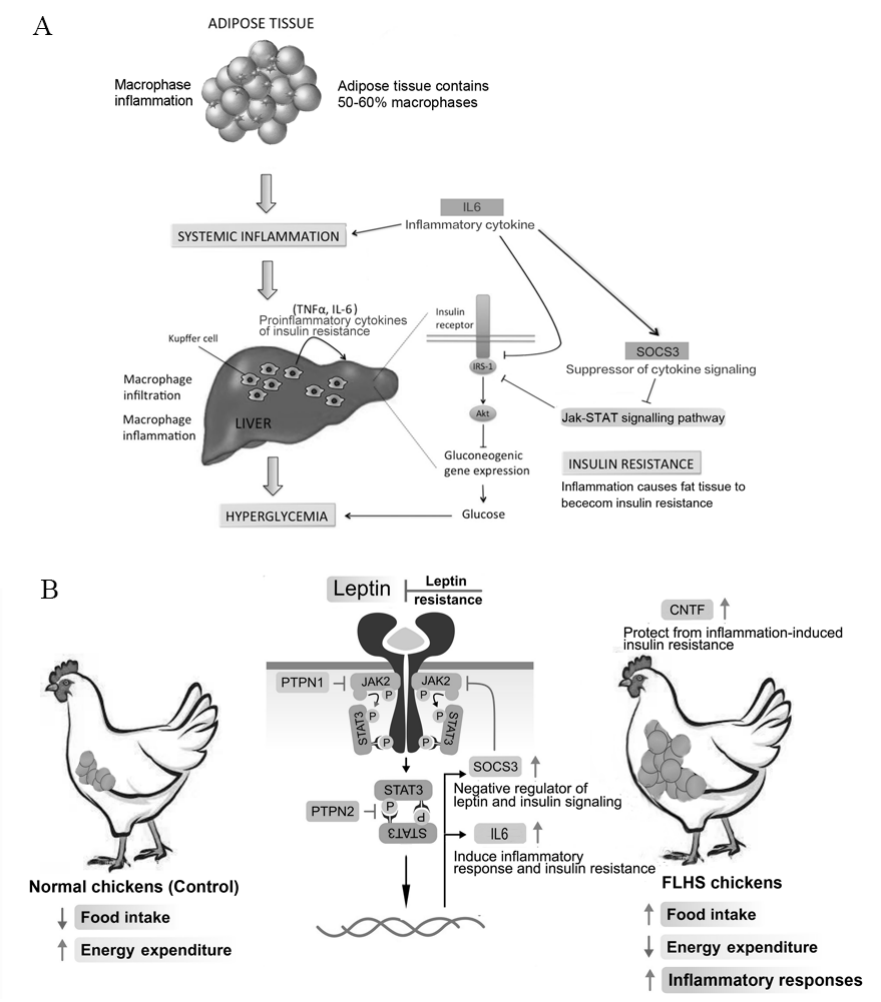
A proposed model of the role of the JAK-STAT signaling pathway in the development of FLHS in chickens. (A) *IL6* and *SOCS3* in macrophages inhibit IRS-1 induced inflammation, hyperglycaemia and insulin resistance. (B) A proposed model of the role of the JAK-STAT signaling pathway in the development of FLHS in chickens. In healthy chickens, leptin induces satiety and increases energy expenditure. While in FLHS affected chickens, the activity of leptin as satiety hormone is decreased due to leptin resistance by suppressor gene of SOC3, inducing the dysfunction of Jak-STAT signaling pathway and resulting in excessive amount of abdominal fat. FLHS, fatty liver hemorrhagic syndrome; *IL-6*, interleukin-6; *SOCS3*, suppressor of cytokine signaling-3; IRS-1, insulin receptor substrate 1.

Then, we tried to understand which biological processes and pathways were overrepresented by all DEGs with a log2(fold change) higher than 1.2 and Padj (false discover rate) less than 0.05 between control and FLHS chickens by DAVID (https://david-d.ncifcrf.gov/) and KEGG database (http://www.genome.jp/kegg/). Two significantly enriched pathways (Jak-STAT signaling pathway and cytokine-cytokine receptor interaction pathway) were identified, including key DEGs of *IL6*, *IL1R2*, *IL13RA2*, *SOCS3*, *CNTF*, and TNF receptor superfamily member 6b which we mentioned before (Table 3). Intriguingly, the JAK-STAT signaling pathway, a landmark in cell biology, is known for regulation of hormones, interferons, colony-stimulating factors, and interleukins [31]. The highly conserved Jak-STAT signaling pathway requests normal homeostasis, and its dysregulation would contribute to the development of obesity, hepatic steatosis and diabetes [32]. On the one hand, *SOCS3*, the up-regulated gene in the pathway in FLHS affected chickens, plays an essential role in mediating inflammatory responses in both immune cells (such as macrophages and T-cells) and metabolic organs (such as the liver, adipose tissue and skeletal muscle), affecting systemic inflammation, insulin resistance, and leptin resistance [6]. As shown in Figure 4A, the important endocrine function of adipose tissue is emphasized by the adverse metabolic consequences of both fat excess and deficiency, associating with macrophage inflammation [33]. Increased inflammation would impair insulin signaling through pro-inflammatory cytokine tumor necrosis factor – alpha (TNFα) and *IL6*, leading to the development of hyper-insulinaemia, accompanied by hepatic insulin resistance, glucose intolerance and reduced insulin signaling in the liver, which is consistent with finding that liver Kupffer cells are a major cause of insulin resistance in obesity [33]. On the other hand, *IL-6* decreases insulin signaling in peripheral tissues by reducing expression of insulin receptor substrate 1 (IRS-1) and inducing *SOCS3* [34,35]. Then *SOCS3* can also reduce IRS1 protein levels through inhibiting Jak-STAT signaling pathway.

Based on the biological functions of above-mentioned genes and previous studies of Jak-STAT signaling pathway, we present a proposed model for the development of chicken FLHS (Figure 4B). In this model, *SOCS3* acts as a negative regulator of the leptin receptor [32]), repressing leptin binding to the receptor and damaging phosphorylation of JAK2, and subsequently, phosphorylation and dimerization of STAT3, which leads to the dysfunction of Jak-STAT signaling pathway and leptin resistance. Leptin is an important hormone synthesized by adipocytes [36], which acts on the hypothalamus to regulate food intake and energy expenditure. Mice lacking either leptin or the leptin receptor (LepRb/ObRb develop severe obesity and insulin resistance. Consistently, in healthy chickens, leptin induces satiety and increases energy expenditure. While in FLHS affected chickens, the activity of leptin as satiety hormone is decreased due to leptin resistance by suppressor gene of *SOC3*, inducing the dysfunction of Jak-STAT signaling pathway and resulting in excessive amount of abdominal fat (Figure 4B). Thereby, these findings suggest that the inhibition of *IL6* and *SOCS3* could potentially serve as a favorable strategy to enhance insulin action and improve glucose homoeostasis. In addition, as obesity related diseases are always associated with hyperactivation of immune cells, efforts to increase the expression of *CNTF* proteins in immune cells may reduce tissue destruction during inflammatory attacks. Its function is like that of leptin, acting centrally by inducing hypothalamic neurogenesis to modulate food intake and peripherally by altering hepatic gene expression (Figure 4). So, the up-regulation of *CNTF* may be a protective mechanism to prevent further deterioration in glucose homoeostasis under conditions of hyperinsulinemia and high nutrient availability, which raises the possibility that *CNTF* has the therapeutic potential for treating obesity-related disorders. Together, better understanding of the JAK-STAT pathway and the functional roles of *IL6*, *SOCS3*, and *CNTF* in the context of FLHS are of importance, which may enable us to develop new and specific therapies for FLHS susceptible commercial laying hens.

We noted that cytokine-cytokine receptor interaction was also associated with inflammatory process (Supplementary Figure S3). Cytokines exert a vast array of immune regulatory actions critical to human biology and disease, which can regulate many important facets of immune function and numerous other aspects of mammalian physiology [35]. DEGs of *IL6*, *IL1R2*, and *CNTF* were also enriched in this pathway, further indicating that immune response and lipid metabolic related genes were implicated in FLHS affected chickens, which is consistent with previous studies in obese humans [32] and fat mice [36]. Collectively, both Jak-STAT signaling pathway and cytokine-cytokine receptor interaction pathway may contribute to the chicken FLHS by regulating inflammatory responses and metabolic homeostasis.

### qPCR and ELISA validation of DEGs enriched in Jak-STAT signaling pathway

**Figure 5:**
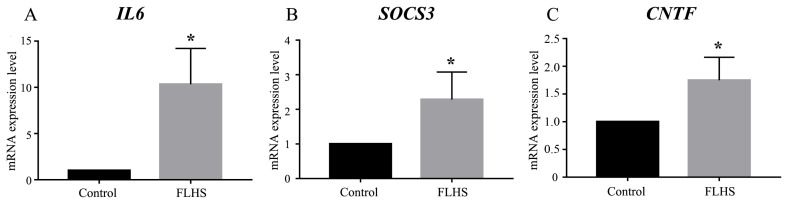
mRNA expression levels of differentially expressed genes revealed by quantitative RT-PCR. (A) *IL6*, (B) *SOCS3*, and (C) *CNTF*. Expression levels in each tissue were determined as the fold-change in 2^−ΔΔCt^ levels relative to the control group with median expression set to 1. RT-PCR, real-time polymerase chain reaction; *IL-6*, interleukin-6; *SOCS3*, suppressor of cytokine signaling-3; *CNTF*, ciliary neurotrophic factor. Significance was assessed by t-test, * p<0.05.

To test the proposed model of JAK-STAT signaling pathway, we further examined whether mRNA expression levels of *IL6*, *SOCS3*, and *CNTF* were elevated using quantitative RT-PCR. We quantified the mRNA expression levels of *IL6*, *SOCS3*, and *CNTF* in FLHS pathological chickens relative to control individuals and normalized the data using β-actin as reference. The mRNA level of *IL6*, *SOCS3*, and *CNTF* were significantly increased by 10.3-fold, 2.3-fold and 1.75-fold (p<0.05) in the FLHS samples, respectively (Figure 5), which are consistent with our RNA-seq data (Table 3). We further conducted ELISA to quantify *IL-6*, *SOCS3*, and *CNTF* protein levels in the circulation. We found that the experimental group had significant higher (p<0.01) levels of the three proteins than the control group (Table 4), which is concordant with the RNA-Seq result that the *IL-6*, *SOCS3*, and *CNTF* mRNA expression was up-regulated in the experimental group.

## CONCLUSION

We present here, to the best of our knowledge, the first comprehensive profile of gene expression in the adipose tissue from healthy chickens and FLHS pathological chickens using RNA sequencing technology. We found that the transcriptome of adipose tissue significantly changed in FLHS affected individuals, which enabled us to identify a list of DEGs (such as *PLA2G5*, *WFIKKN2*, *DEGS2*, *PLIN3*, *IL6*, *CNTF*) and their enriched biological processes and pathways. By integrating with previous reports, we then proposed a reasonable model to explain the genetic mechanisms underlying FLHS in chickens and further highlighted *IL6*, *SOCS3*, and *CNTF* in the Jak-STAT signaling pathway as potential therapeutic targets for chicken FLHS, though further studies are needed to confirm the proposed model and highlighted candidate genes for the FLHS disease in laying hens.

## Figures and Tables

**Table 1:** Effect of fatty liver hemorrhagic syndrome on liver index, hepatic triglyceride and hepatic total cholesterol in chickens

Parameters	Control	Experimental
Liver index1) (‰)	14.12±0.70^A^	20.03±0.68^B^
Hepatic TG (mmol/g)	4.22±0.29^A^	9.44±0.43^B^
Hepatic TC (mmol/g)	1.64±0.15^A^	3.30±0.31^B^

Values are presented as the mean±standard deviation (n≥10).

TG, triglyceride; TC, total cholesterol.

1) Liver index (‰) = humid weight of liver/body weight.

AB Values with different superscripts show highly significant difference between different groups (p<0.01).

**Table 2:** Top 10 up- and down- regulated genes in FLHS affected chickens

Gene	log2(fold change)	p-value	Padj	Regulate
*PLA2G5*	7.03	6.89E-04	4.99E-02	Up
*DEGS2*	5.91	1.48E-04	1.89E-02	Up
*RSPO4*	5.17	6.13E-07	5.72E-04	Up
*GGCL1*	4.50	1.14E-08	2.28E-05	Up
*IL6*	4.36	5.55E-07	5.55E-04	Up
*SLC9A2*	3.87	4.63E-04	3.92E-02	Up
*SLITRK4*	3.65	7.88E-06	3.34E-03	Up
*ATP6V0D2*	3.47	8.80E-06	3.62E-03	Up
*IL13RA2*	3.18	5.75E-06	2.76E-03	Up
*EPHA1*	3.16	5.74E-05	1.07E-02	Up
*WFIKKN2*	−2.73	1.54E-04	1.94E-02	Down
*RD3L*	−1.91	1.33E-05	4.55E-03	Down
*PLIN3*	−1.87	5.62E-04	4.39E-02	Down
*PIANP*	−1.58	4.64E-04	3.92E-02	Down
*PLPP1*	−1.49	9.44E-05	1.43E-02	Down
*RFESD*	−1.48	8.31E-05	1.34E-02	Down
*TMEM26*	−1.40	3.03E-11	4.25E-07	Down
*LRRTM4*	−1.37	3.39E-04	3.24E-02	Down
*RASSF9*	−1.29	9.36E-05	1.43E-02	Down

*PLA2G5*, phospholipase A2 group V; *DEGS2*, delta 4-desaturase sphingolipid 2; *RSPO4*, R-Spondin 4; *GGCL1*, gallus chemokine-like ligand 1; *IL6*, interleukin-6; *SLC9A2*, solute carrier family 9 member 2; *SLITRK4*, slit and NTRK like family member 4; *ATP6V0D2*, ATPase H+ Transporting V0 subunit d2; *IL13RA2*, interleukin 13 receptor subunit alpha 2; *EPHA1*, eph receptor a1; *WFIKKN2*, WAP kunitz and netrin domain containing 2; *RD3L*, retinal degeneration 3 like; *PLIN3*, perilipin 3; *PIANP*, PILR alpha associated neural protein; *PLPP1*, phospholipid phosphatase 1; *RFESD*, RIESKE FE-S domain containing; *TMEM26*, transmembrane protein 26; *LRRTM4*, leucine rich repeat transmembrane neuronal 4; *RASSF9*, Ras association domain family member 9.

**Table 3:** Top canonical pathways that are enriched by differentially expressed genes between FLHS affected and non-affected chickens

Pathway	Genes	log2(Fold Change)	p-value	Padj	Regulate	Function
gga04630:Jak-STAT signaling pathway	*IL6*	4.36	5.55E-07	5.55E-04	Up	It acts as a cytokine that functions in inflammation and the maturation of B cells.
	*IL13RA2*	3.18	5.75E-06	2.76E-03	Up	It is a cytokine secreted by activated T lymphocytes that shares many logical activities with IL-4.
	*SOCS3*	2.13	1.35E-04	1.80E-02	Up	It inhibits the functioning of leptin and downstream steps in insulin signaling after being expressed by terminal transcription factors, such as STAT3 and c-fos.
	*CNTF*	1.54	1.05E-07	1.63E-04	Up	The protein is a potent survival factor for neurons and oligodendrocytes and may be relevant in reducing tissue destruction during inflammatory attacks.
gga04060:Cytokine-cytokine receptor interaction	*IL6*	4.36	5.55E-07	5.55E-04	Up	It acts as a cytokine that functions in inflammation and the maturation of B cells.
	*TNFRSF6B*	2.03	7.43E-05	1.21E-02	Up	It acts as a decoy receptor that competes with death receptors for ligand binding.
	*IL1R2*	1.76	2.94E-04	3.06E-02	Up	The protein can bind interleukin alpha (IL1A), interleukin beta (IL1B), and interleukin 1 receptor, type I (IL1R1/IL1RA), and acts as a decoy receptor that inhibits the activity of its ligands.
	*CNTF*	1.54	1.05E-07	1.63E-04	Up	The protein is a potent survival factor for neurons and oligodendrocytes and may be relevant in reducing tissue destruction during inflammatory attacks.

*IL6*, interleukin-6; *IL13RA2*, interleukin 13 receptor subunit alpha 2; *SOCS3*, suppressor of cytokine signaling 3; *CNTF*, ciliary neurotrophic factor; *TNFRSF6B*, TNF receptor superfamily member 6b; *IL1R2*, interleukin 1 receptor, type II.

**Table 4:** Serum *IL-6*, *CNTF*, and *SOCS3* contents in healthy chickens and FLHS affected chickens

Genes	Control group	FLHS group
*IL-6* (pg/mL)	3.12±1.38^A^	17.45±7.29^B^
*SOCS3* (pg/mL)	44.81±11.64^A^	126.94±29.03^B^
*CNTF* (pg/mL)	6.00±2.72^A^	80.23±21.56^B^

Values are presented as the mean±standard deviation (n≥10).

*IL-6*, interleukin-6; *CNTF*, ciliary neurotrophic factor; *SOCS3*, suppressor of cytokine signaling-3; FLHS, fatty liver hemorrhagic syndrome.

AB Values with different superscripts show highly significant difference between different groups (p<0.01).
