# Supplementary material for: Effect of JAK-STAT pathway in regulation of fatty liver hemorrhagic syndrome in chickens
Source: Anim Biosci. 2020 Feb 25;34(1):143–53. doi: 10.5713/ajas.19.0874 (PMC7888496; doi:10.5713/ajas.19.0874)
Supplement: Supplementary file 1 [file ajas-19-0874-suppl.pdf]

SUPPLEMENTARY MATERIAL

Effect of JAK-STAT pathway in regulation of fatty liver hemorrhagic syndrome in chickens

The PDF file includes:

Four supplementary tables: Table S1 - S4

Four supplementary figures: Figure S1 – S4

Table S1. Ingredient and nutrient levels of the HELP diets in control and experimental group

Table S2. The 100 up- and down- regulated genes in FLHS chicken.

Table S3. The related biological functions of the 66 DEGs.

Table S4. Gene ontology (GO) enriched by differentially expressed genes between FLHS affected and non-affected chickens.

Figure S1. Summary of RNA-Seq data. (A) The total number of sequence reads for each sample. (B) Average mapping statistics and proportion of reads mapping to exon, intergenic and intron. CTR, control; FLHS, Fatty liver hemorrhagic syndrome.

Figure S2. The volcano plot of differential expressed genes between CTR and FLHS chickens. The red and blue dot indicates up regulated gene and down regulated gene, respectively.

Figure S3. RNA-Seq bioinformatics workflow showing analyze steps (light blue) and functional results (light green).

Figure S4. Pathway of cytokine-cytokine receptor interaction.

Table S1. Ingredient and nutrient levels of the HELP diets in control and experimental group

HELP diet		Control group (%)	Experimental group (%)
Ingredient	Corn	64	70
	Wheat bran	2	1.2
	Soybean meal	24	14.58
	Fat-soybean oil	-	4.22
	Limestone	8	8
	Premix*	2	2
Nutrient levels	Energy (Mcal/kg)	11.21	12.97
	Crude protein (%)	15.86	12
	P (%)	0.51	0.46
	Arg (%)	1.03	0.74
	Met (%)	0.37	0.32
	Val (%)	0.77	0.58
	Met + Cys (%)	0.67	0.56

*Premix was composed of the following per kg diet: Cu, 2.50 mg; Fe, 20.00 mg; Zn, 17.50 mg; Mn, 15.00 mg; KI, 4.00 mg; Na2SeO3, 6.00 mg; CoCl, 2.50 mg; Met, 50.00 mg; pyridine chromium, 2.00 mg; vitamins, 15.00 mg; phytase, 10.00 mg; kininase, 7.50 mg; antioxidant, 2.00 mg; lycine, 15.00 mg; choline, 50.00 mg; salt, 200.00 mg; Ca3PO4, 500.00 mg and zeolite, 76.00 mg.

Table S2. The 100 up and down regulated DEGs in FLHS chicken.

Gene	log2(FoldChange)	P-value	Padj	Regulate
PLA2G5	7.03	6.89E-04	4.99E-02	Up
DEGS2	5.91	1.48E-04	1.89E-02	Up
MSTRG.14196	-5.76	3.24E-05	6.97E-03	Down
RSPO4	5.17	6.13E-07	5.72E-04	Up
MSTRG.10070	5.10	1.32E-04	1.78E-02	Up
MSTRG.7302	4.63	2.39E-04	2.66E-02	Up
GGCL1	4.50	1.14E-08	2.28E-05	Up
IL6	4.36	5.55E-07	5.55E-04	Up
MSTRG.6350	3.97	5.87E-04	4.52E-02	Up
MSTRG.3432	3.92	4.17E-09	9.72E-06	Up
SLC9A2	3.87	4.63E-04	3.92E-02	Up
SLITRK4	3.65	7.88E-06	3.34E-03	Up
ATP6V0D2	3.47	8.80E-06	3.62E-03	Up
MSTRG.12924	3.24	3.61E-05	7.54E-03	Up
IL13RA2	3.18	5.75E-06	2.76E-03	Up
MSTRG.8644	3.18	5.14E-04	4.16E-02	Up
EPHA1	3.16	5.74E-05	1.07E-02	Up
RRAD	3.05	7.23E-05	1.19E-02	Up

MSTRG.7990	2.95	7.05E-10	2.47E-06	Up
NR4A2	2.87	5.20E-05	9.98E-03	Up
LGALS2	2.86	1.69E-04	2.06E-02	Up
NCF2	2.83	9.89E-06	3.83E-03	Up
WFIKKN2	-2.73	1.54E-04	1.94E-02	Down
ADAMTS4	2.52	2.01E-07	2.82E-04	Up
KCNQ4	2.48	1.44E-05	4.71E-03	Up
MSMB	2.45	1.59E-04	1.97E-02	Up
il-1beta	2.32	7.03E-05	1.17E-02	Up
MSTRG.11611	2.30	2.29E-05	5.78E-03	Up
MSTRG.4071	2.28	7.13E-06	3.12E-03	Up
CCL4	2.27	5.01E-04	4.10E-02	Up
SLC11A1	2.26	4.72E-04	3.96E-02	Up
CCLi8	2.25	1.91E-04	2.23E-02	Up
MSTRG.11750	2.22	1.73E-04	2.08E-02	Up
CD9	2.20	3.29E-06	2.07E-03	Up
MSTRG.9375	2.17	3.29E-04	3.24E-02	Up
IRG1	2.15	3.21E-04	3.24E-02	Up
MSTRG.4815	2.14	5.27E-07	5.55E-04	Up
SOCS3	2.13	1.35E-04	1.80E-02	Up
MSTRG.14322	-2.08	2.08E-04	2.35E-02	Down
ANGPTL4	2.06	1.24E-04	1.76E-02	Up
SULF1	2.05	2.86E-06	1.91E-03	Up
NTN1	2.05	6.48E-05	1.14E-02	Up
SMOC1	2.04	2.83E-04	3.01E-02	Up
TNFRSF6B	2.03	7.43E-05	1.21E-02	Up
ZC3H12D	2.02	6.91E-04	4.99E-02	Up
MSTRG.11420	-2.01	3.09E-05	6.86E-03	Down
RAB7B	1.96	1.05E-04	1.54E-02	Up
TNC	1.94	6.95E-05	1.17E-02	Up
EGR3	1.92	1.74E-04	2.08E-02	Up
RD3L	-1.91	1.33E-05	4.55E-03	Down
MSTRG.4809	1.89	1.02E-04	1.50E-02	Up
PLIN3	-1.87	5.62E-04	4.39E-02	Down
TLR15	1.87	1.40E-05	4.66E-03	Up
MSTRG.11285	1.79	1.60E-04	1.97E-02	Up
CCLi7	1.78	6.86E-05	1.17E-02	Up
IGFBP2	1.77	1.17E-04	1.67E-02	Up
IL1R2	1.76	2.94E-04	3.06E-02	Up
MSTRG.8076	1.74	4.64E-04	3.92E-02	Up
EGR2	1.70	2.08E-04	2.35E-02	Up
MSTRG.7401	-1.69	2.46E-04	2.69E-02	Down
CLIC3	1.69	4.56E-10	2.13E-06	Up
CDKN1A	1.66	3.50E-04	3.29E-02	Up

MSTRG.14286	1.66	3.86E-04	3.51E-02	Up
MSTRG.6324	1.64	3.14E-04	3.21E-02	Up
PIANP	-1.58	4.64E-04	3.92E-02	Down
MSTRG.1199	1.57	3.40E-06	2.07E-03	Up
CAPN3	1.56	2.85E-04	3.01E-02	Up
MSTRG.8077	1.56	4.60E-04	3.92E-02	Up
CNTF	1.54	1.05E-07	1.63E-04	Up
NPM3	1.53	1.57E-09	4.39E-06	Up
MSTRG.1068	1.52	1.60E-06	1.25E-03	Up
MSTRG.9972	1.51	2.95E-04	3.06E-02	Up
MSTRG.12156	1.50	7.83E-07	6.86E-04	Up
PLPP1	-1.49	9.44E-05	1.43E-02	Down
RFESD	-1.48	8.31E-05	1.34E-02	Down
EAF2	1.45	6.09E-04	4.61E-02	Up
MSTRG.8767	-1.43	3.65E-04	3.38E-02	Down
HSPB8	1.43	5.31E-05	1.01E-02	Up
MSTRG.6239	1.43	3.84E-04	3.51E-02	Up
LRRC17	-1.43	5.36E-06	2.68E-03	Down
MSTRG.458	-1.42	2.84E-08	4.97E-05	Down
TMEM26	-1.40	3.03E-11	4.25E-07	Down
PTPRF	1.39	5.49E-04	4.32E-02	Up
MSTRG.7281	1.39	2.47E-07	3.14E-04	Up
gga-mir-6645	1.39	1.36E-04	1.80E-02	Up
HBEGF	1.38	3.49E-10	2.13E-06	Up
LRRTM4	-1.37	3.39E-04	3.24E-02	Down
ACKR4	1.37	5.91E-06	2.76E-03	Up
RHOG	1.36	3.09E-04	3.18E-02	Up
KCNE3	1.35	1.01E-05	3.83E-03	Up
MSTRG.13289	1.34	1.45E-04	1.89E-02	Up
ARL4C	1.34	9.57E-07	7.89E-04	Up
RASSF9	-1.29	9.36E-05	1.43E-02	Down
CHRD	1.28	9.38E-05	1.43E-02	Up
MSTRG.4797	1.26	4.25E-06	2.29E-03	Up
CKB	1.26	1.32E-04	1.78E-02	Up
RTN4RL2	1.25	1.59E-04	1.97E-02	Up
MSTRG.7202	1.25	4.33E-04	3.84E-02	Up
SLC52A3	1.21	4.17E-06	2.29E-03	Up
MSTRG.6051	-1.20	1.64E-05	5.21E-03	Down

Table S3. The related biological functions of the 66 DEGs.

Gene	Symbol	Biology process
DEGS2	Uncharacterized protein	Biosynthetic process(GO:0009058);cellular process(GO:0009987);lipid metabolic process(GO:0006629);nitrogen compound metabolic process(GO:0006807)
IRG1	Uncharacterized protein	Cellular process(GO:0009987);fatty acid metabolic process(GO:0006631);response to stress(GO:0006950)
CCL4	C-C motif chemokine 4 homolog	G-protein coupled receptor signaling pathway(GO:0007186);MAPK cascade(GO:0000165);catabolic process(GO:0009056);cellular component movement(GO:0006928);cytokine-mediated signaling pathway(GO:0019221);localization(GO:0051179);locomotion(GO:0040011);nitrogen compound metabolic process(GO:0006807);regulation of catalytic activity(GO:0050790);regulation of nucleobase-containing compound metabolic process(GO:0019219);regulation of phosphate metabolic process(GO:0019220);response to external stimulus(GO:0009605);Inflammation mediated by chemokine and cytokine signaling pathway
CCLi7	Chemokine	G-protein coupled receptor signaling pathway(GO:0007186);MAPK cascade(GO:0000165);catabolic process(GO:0009056);cellular component movement(GO:0006928);cytokine-mediated signaling pathway(GO:0019221);localization(GO:0051179);locomotion(GO:0040011);nitrogen compound metabolic process(GO:0006807);regulation of catalytic activity(GO:0050790);regulation of nucleobase-containing compound metabolic process(GO:0019219);regulation of phosphate metabolic process(GO:0019220);response to external stimulus(GO:0009605);response to interferon-gamma(GO:0034341);response to stress(GO:0006950);Inflammation mediated by chemokine and cytokine signaling pathway

CCLi8	Chemokine	G-protein coupled receptor signaling pathway(GO:0007186);MAPK cascade(GO:0000165);catabolic process(GO:0009056);cytokine-mediated signaling pathway(GO:0019221);localization(GO:0051179);locomotion(GO:0040011);nitrogen compound metabolic process(GO:0006807);regulation of catalytic activity(GO:0050790);regulation of nucleobase-containing compound metabolic process(GO:0019219);regulation of phosphate metabolic process(GO:0019220);response to external stimulus(GO:0009605);response to interferon-gamma(GO:0034341);response to stress(GO:0006950);Inflammation mediated by chemokine and cytokine signaling pathway
WFIKKN2	Uncharacterized protein	Immune system process(GO:0002376)
SLC11A1	Natural resistance-associated macrophage protein 1	Immune system process(GO:0002376);response to stress(GO:0006950)
CDKN1A	Uncharacterized protein	Interleukin signaling pathway
PLIN3	Perilipin	Lipid metabolic process(GO:0006629)
RFESD	Uncharacterized protein	Metabolic process(GO:0008152)
IL6	Interleukin-6	Inflammation mediated by chemokine and cytokine signaling pathway
ANGPTL4	Uncharacterized protein	Cell surface receptor signaling pathway(GO:0007166);cell-cell adhesion(GO:0016337);cell-matrix adhesion(GO:0007160)
ACKR4	Uncharacterized protein	Apoptotic process(GO:0006915);cellular process(GO:0009987);regulation of biological process(GO:0050789)
EGR2	Uncharacterized protein	Biosynthetic process(GO:0009058);cellular process(GO:0009987);nitrogen compound metabolic process(GO:0006807)
NR4A2	Uncharacterized protein	Biosynthetic process(GO:0009058);cellular process(GO:0009987);nitrogen compound metabolic process(GO:0006807);response to endogenous stimulus(GO:0009719)

RHOG	Uncharacterized protein	Catabolic process(GO:0009056);cellular component morphogenesis(GO:0032989);cytoskeleton organization(GO:0007010);intracellular signal transduction(GO:0035556);nitrogen compound metabolic process(GO:0006807);nucleobase-containing compound metabolic process(GO:0006139);phosphate-containing compound metabolic process(GO:0006796);regulation of biological process(GO:0050789);response to stimulus(GO:0050896);EGF receptor signaling pathway
ATP6V0D2	V-type proton ATPase subunit d 2	Catabolic process(GO:0009056);cellular process(GO:0009987);homeostatic process(GO:0042592);nitrogen compound metabolic process(GO:0006807);nucleobase-containing compound metabolic process(GO:0006139);phosphate-containing compound metabolic process(GO:0006796)
RAB7B	Uncharacterized protein	Catabolic process(GO:0009056);lysosomal transport(GO:0007041);nitrogen compound metabolic process(GO:0006807);nucleobase-containing compound metabolic process(GO:0006139);organelle organization(GO:0006996);phagocytosis(GO:0006909);phosphate-containing compound metabolic process(GO:0006796);protein localization(GO:0008104);regulation of biological process(GO:0050789);response to endogenous stimulus(GO:0009719)
HBEGF	Proheparin-binding EGF-like growth factor	CCKR signaling pathway
CD9	Tetraspanin	Cell surface receptor signaling pathway(GO:0007166);regulation of biological process(GO:0050789);response to stimulus(GO:0050896)
IL1R2	Uncharacterized protein	Cellular process(GO:0009987)
LGALS2	Galectin	Cellular process(GO:0009987)
NPM3	Uncharacterized protein	Cellular process(GO:0009987);chromatin organization(GO:0006325);chromatin remodeling(GO:0006338)
NTN1	Netrin-1	Cellular process(GO:0009987);ectoderm development(GO:0007398);neurological system process(GO:0050877)
SLC9A2	Sodium/hydrogen exchanger	Cellular process(GO:0009987);homeostatic process(GO:0042592)

KCNE3	Uncharacterized protein	Cellular process(GO:0009987);homeostatic process(GO:0042592);regulation of biological process(GO:0050789)
PTPRF	Uncharacterized protein	Cellular protein modification process(GO:0006464);cytoplasm(GO:0005737)
SLC52A3	Uncharacterized protein	G-protein coupled receptor activity(GO:0004930)
ARL4C	Uncharacterized protein	G-protein coupled receptor signaling pathway(GO:0007186)
EPHA1	Uncharacterized protein	MAPK cascade(GO:0000165);cell differentiation(GO:0030154);negative regulation of apoptotic process(GO:0043066);regulation of catalytic activity(GO:0050790);regulation of phosphate metabolic process(GO:0019220);response to stimulus(GO:0050896);single-multicellular organism process(GO:0044707);transmembrane receptor protein tyrosine kinase signaling pathway(GO:0007169)
RRAD	Uncharacterized protein	Neurological system process(GO:0050877);synaptic transmission(GO:0007268)
ZC3H12D	Uncharacterized protein	Nucleic acid binding(GO:0003676)
SULF1	Uncharacterized protein	Phospholipid metabolic process(GO:0006644);polysaccharide metabolic process(GO:0005976);sulfur compound metabolic process(GO:0006790)
PLA2G5	Phospholipase A(2)	Phospholipid metabolic process(GO:0006644);signal transduction(GO:0007165)
CAPN3	Calpain-3	Proteolysis(GO:0006508)
IGFBP2	Insulin-like growth factor-binding protein 2	Regulation of biological process(GO:0050789);response to stimulus(GO:0050896);transmembrane receptor protein tyrosine kinase signaling pathway(GO:0007169)
IL13RA2	Uncharacterized protein	Response to stimulus(GO:0050896)
NCF2	Uncharacterized protein	Response to stimulus(GO:0050896)
TLR15	Uncharacterized protein	Toll receptor signaling pathway
RD3L	Uncharacterized protein	No data
RASSF9	Uncharacterized protein	No data
EAF2	ELL-associated factor 2	No data

SMOC1	Uncharacterized protein	No data
HSPB8	Uncharacterized protein	No data
PIANP	Uncharacterized protein	No data
TNFRSF6B	Uncharacterized protein	No data
CKB	Creatine kinase B-type	No data
TMEM26	Uncharacterized protein	No data
SLITRK4	Uncharacterized protein	No data
6629	Interleukin-6	No data
CNTF	Ciliary neurotrophic factor	No data
TNC	Tenascin	No data
SOCS3	Suppressor of cytokine signaling 3	No data
ADAMTS4	Uncharacterized protein	No data
CHRD	Uncharacterized protein	No data
CLIC3	Uncharacterized protein	No data
EGR3	Uncharacterized protein	No data
gga-mir-6645	Uncharacterized protein	No data
GGCL1	Uncharacterized protein	No data
il-1beta	Uncharacterized protein	No data
KCNQ4	Uncharacterized protein	No data
LRRC17	Uncharacterized protein	No data
LRRTM4	Uncharacterized protein	No data
MSMB	Uncharacterized protein	No data
PLPP1	Uncharacterized protein	No data
RSPO4	Uncharacterized protein	No data
RTN4RL2	Uncharacterized protein	No data

Table S4. Gene ontology (GO) enriched by differentially expressed genes between FLHS affected and non-affected chickens.

Gene Ontology	Counts	P-value	Genes
GO:0044421~extracellular region part	7	7.13E-04	*IL6*, *CNTF*, *TNC*, *HBEGF*, *IGFBP2*, *NTN1*, *ANGPTL4*
GO:0005615~extracellular space	5	0.00269	*IL6*, *CNTF*, *HBEGF*, *IGFBP2*, *ANGPTL4*
GO:0005576~extracellular region	8	0.00310	*WFIKKN2*, *IL6*, *CNTF*, *TNC*, *HBEGF*, *IGFBP2*, *NTN1*, *ANGPTL4*
GO:0051146~striated muscle cell differentiation	3	0.00714	*TNC*, *NTN1*, *CAPN3*
GO:0006811~ion transport	6	0.01055	*SLC11A1*, *KCNQ4*, *CLIC3*, *SLC9A2*, *ATP6V0D2*, *KCNE3*
GO:0005138~interleukin-6 receptor binding	2	0.01085	*IL6*, *CNTF*
GO:0042692~muscle cell differentiation	3	0.01399	*TNC*, *NTN1*, *CAPN3*
GO:0065007~biological regulation	15	0.01539	*IL6*, *TLR15*, *CNTF*, *TNC*, *SLC9A2*, *RRAD*, *NTN1*, *CD9*, *SLC11A1*, *CNTF*, *HBEGF*, *EAF2*, *IGFBP2*, *RHOG*, *ANGPTL4*
GO:0009653~anatomical structure morphogenesis	5	0.04438	*CD9*, *IL6*, *CNTF*, *NTN1*, *CAPN3*
GO:0042592~homeostatic process	4	0.04451	*SLC11A1*, *CD9*, *IL6*, *SLC9A2*
GO:0005244~voltage-gated ion channel activity	3	0.04596	*KCNQ4*, *CLIC3*, *KCNE3*
GO:0022832~voltage-gated channel activity	3	0.04596	*KCNQ4*, *CLIC3*, *KCNE3*
GO:0015491~cation:cation antiporter activity	2	0.04623	*SLC11A1*, *SLC9A2*
GO:0050801~ion homeostasis	3	0.04712	*SLC11A1*, *CD9*, *SLC9A2*
GO:0042127~regulation of cell proliferation	4	0.04752	*CD9*, *IL6*, *CNTF*, *HBEGF*
GO:0010927~cellular component assembly involved in morphogenesis	2	0.04890	*CD9*, *CAPN3*
GO:0050794~regulation of cellular process	13	0.04891	*IL6*, *TLR15*, *CNTF*, *TNC*, *RRAD*, *NTN1*, *CD9*, *CNTF*, *HBEGF*, *EAF2*, *IGFBP2*, *RHOG*, *ANGPTL4*
GO:0015299~solute:hydrogen antiporter activity	2	0.04970	*SLC11A1*, *SLC9A2*

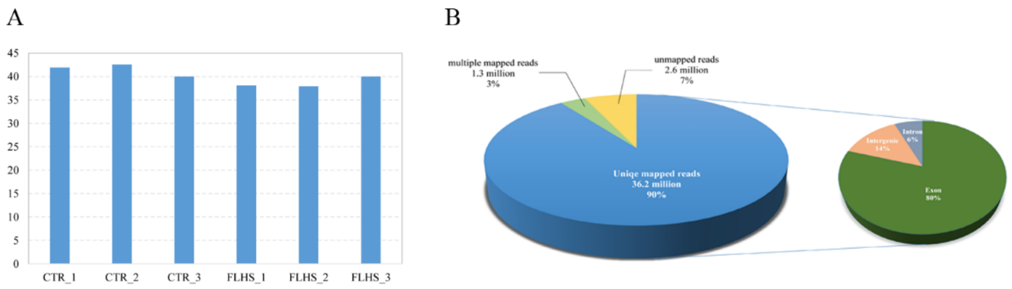

Figure S1. Summary of RNA-Seq data. (A) The total number of sequence reads for each sample. (B) Average mapping statistics and proportion of reads mapping to exon, intergenic and intron. CTR, control; FLHS, Fatty liver hemorrhagic syndrome.

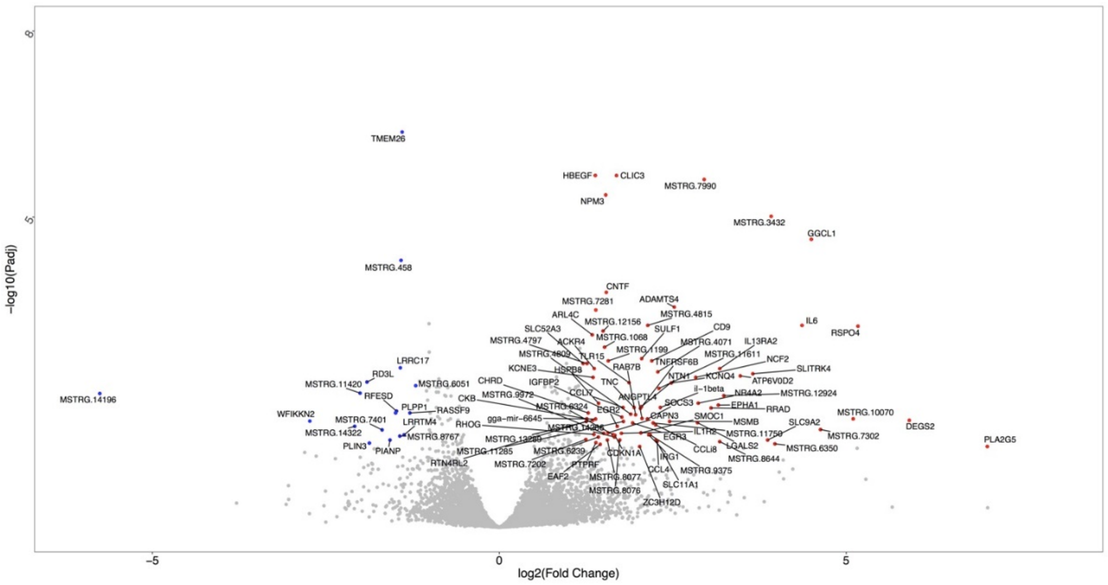

Figure S2. The volcano plot of differential expressed genes between CTR and FLHS chickens. The red and blue dot indicates up regulated gene and down regulated gene, respectively.

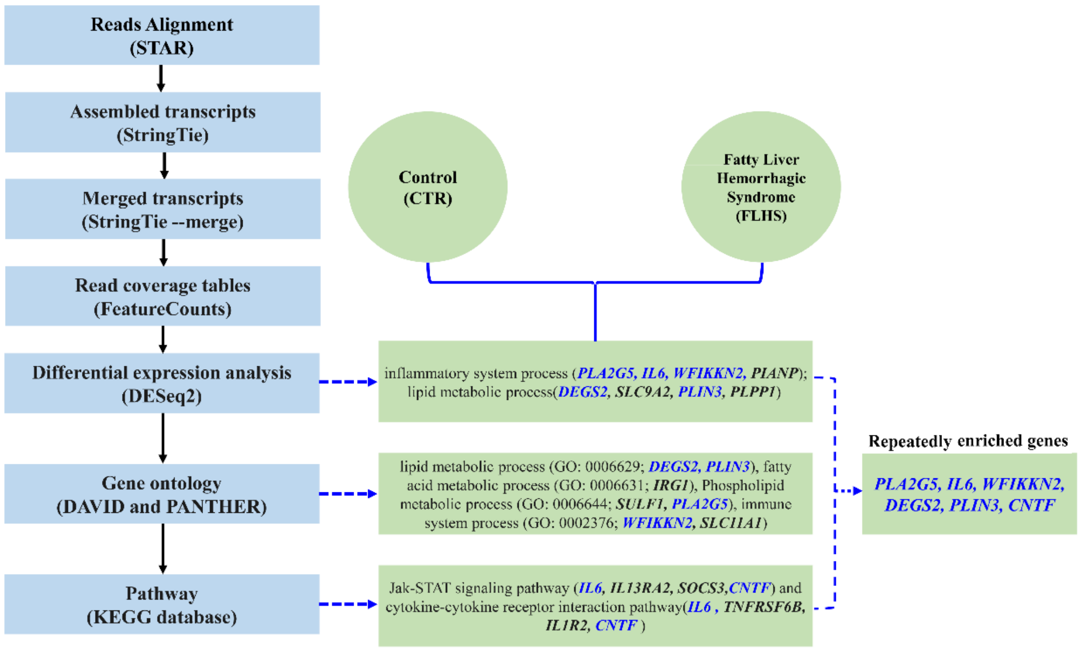

Figure S3. RNA-Seq bioinformatics workflow showing analyze steps (light blue) and functional results (light green).

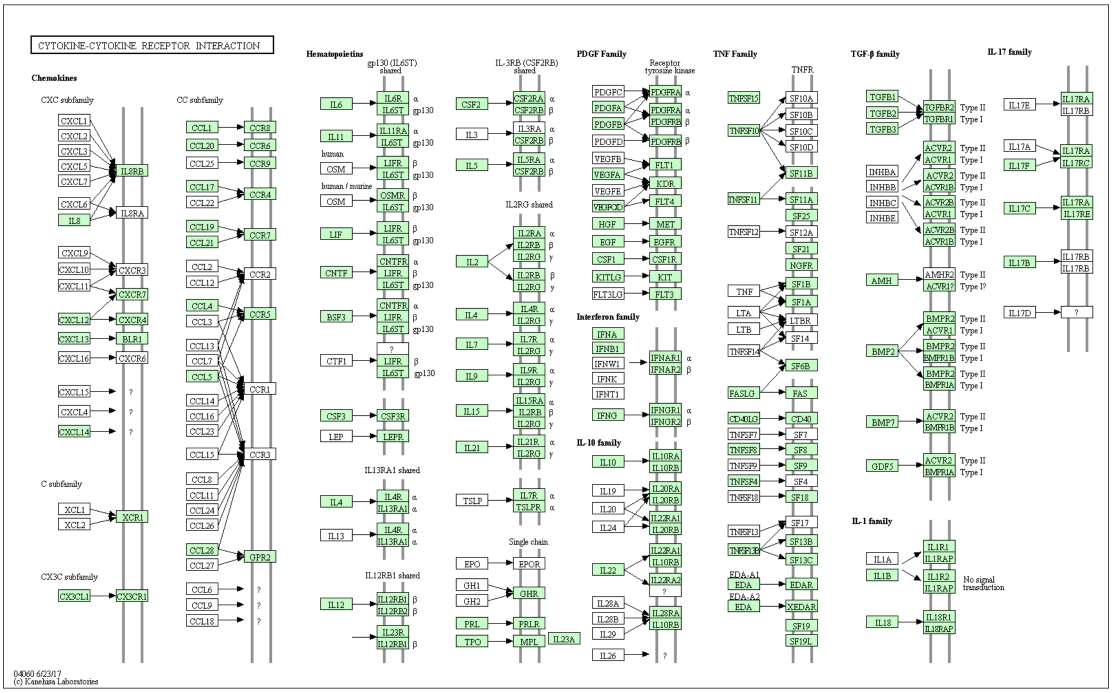

Figure S4. Pathway of cytokine-cytokine receptor interaction.
